# Collision Tumour of the Ovary: Mature Cystic Teratoma and Benign Mucinous Cystadenoma

**DOI:** 10.7759/cureus.106548

**Published:** 2026-04-06

**Authors:** Jia Ni Kwan, Anusha Sudhahar

**Affiliations:** 1 Department of Obstetrics and Gynaecology, Ipswich Hospital, Ipswich, AUS

**Keywords:** benign mucinous cystadenoma, collision tumour, dermoid, mature cystic teratoma, ovary

## Abstract

A collision tumour is defined as the coexistence of two or more histologically and pathogenetically distinct tumours without any histological intermixing in the same organ or tissue. This article reports a case of an ovarian collision tumour consisting of a mature cystic teratoma and a benign mucinous cystadenoma in a 28-year-old premenopausal woman. Ultrasound of the pelvis showed an enlarged right ovary with a 7.0 cm x 6.0 cm x 5.0 cm cyst. The tumour marker was normal. Macroscopic examination of the cyst revealed a dermoid cyst with some haemorrhagic areas. The histopathological examination showed a dermoid cyst that matched the characteristics of a mature cystic teratoma and a benign mucinous cystadenoma, both located in the right ovary. Recognition of collision tumours by gynaecologists, radiologists, and pathologists is vital. Detailed histopathological evaluation is essential for accurate diagnosis and for tailoring appropriate therapy according to the distinct biological behaviour of the individual tumour components.

## Introduction

Ovarian tumours represent a heterogeneous group of neoplasms with diverse histogenesis and biological behaviour. They encompass a wide spectrum of neoplasms arising from epithelial, germ cell, and sex cord-stromal origins. Epithelial tumours are the most common, including serous, mucinous, endometrioid, clear cell, and transitional cell subtypes. Germ cell tumours include teratomas (mature and immature), and sex cord-stromal tumours include granulosa cell tumours and fibromas [1]. 

A collision tumour is defined as the coexistence of two or more histologically and pathogenetically distinct tumours without any histological intermixing in the same organ or tissue. Collision tumours have been described in many different types of organs such as the gastrointestinal tract, lung, skin, adrenal, central nervous system, lymph node, and uterus. However, it is distinctly rare to have a collision tumour of the ovary [2]. The first reported case of an ovarian collision tumour was described by Copland and Coleman in 1946, with subsequent literature consisting largely of isolated case reports [3]. The incidence of ovarian collision tumour is unclear, as most knowledge comes from case reports and small case series.

We present a rare case of an ovarian collision tumour comprising a mature cystic teratoma and a benign mucinous cystadenoma, highlighting its clinical presentation, radiological findings, and histopathological features. Increased awareness of this uncommon entity may aid clinicians and pathologists in accurate diagnosis and appropriate management.

## Case presentation

A 28-year-old nulliparous woman was referred by her general practitioner to the gynaecology clinic for assessment of a right ovarian cyst. She presented with a history of menorrhagia and mild dysmenorrhoea. Menarche occurred at 11 years of age, but her menstrual cycles had been irregular, characterised by heavy bleeding, typically lasting three days. She denied urinary or bowel symptoms. Her most recent cervical screening test was normal, and she had no history of sexually transmitted infections. She had anxiety and depression, for which she was being treated with reboxetine and lorazepam. On examination, she was a well-looking woman with a body mass index (BMI) of 30 kg/m². Abdominal examination revealed a soft, non-tender abdomen with no palpable masses. Pelvic examination was not performed.

She initially underwent a computed tomography (CT) scan for investigation of lower back pain, which demonstrated a central disc protrusion at the L4-L5 level. An incidental finding of a 5.7 cm fat-containing right adnexal lesion was also noted. Subsequently, a pelvic ultrasound was performed. The right ovary was enlarged, with a calculated volume of 114 cc. Within the ovary, there was an avascular echogenic lesion measuring 7.0 cm × 6.0 cm × 5.0 cm, consistent with a right ovarian dermoid cyst. Adjacent to the right ovary, an elongated septated cystic structure measuring 4.0 cm × 2.7 cm × 2.4 cm was identified, raising suspicion of a right hydrosalpinx. The endometrium appeared thickened, measuring 15 mm. Serum tumour marker testing showed a normal cancer antigen 125 (CA-125) level of 19 U/mL; no other tumour markers were assessed.

**Figure 1:**
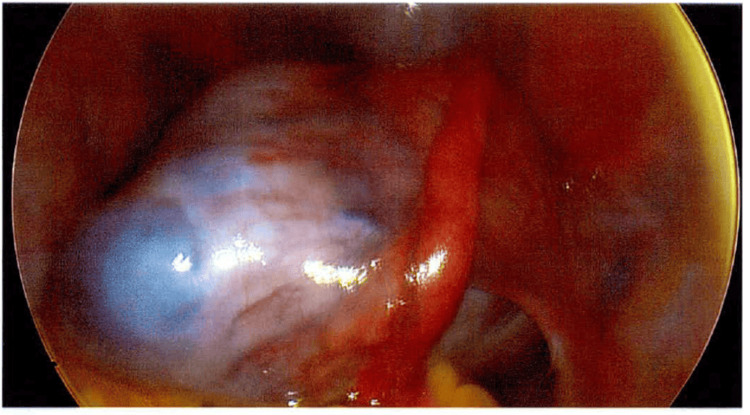
A large right ovarian cyst was noted during laparoscopy

The patient subsequently underwent hysteroscopy with dilatation and curettage, laparoscopic right ovarian cystectomy, and insertion of a levonorgestrel-releasing intrauterine system (Mirena). Intraoperatively, a large right ovarian cyst measuring approximately 10.0 cm was identified (Figure 1). The cyst wall was opened using monopolar diathermy. During initial dissection, inadvertent cyst rupture occurred, with extrusion of sebaceous material and hair, consistent with a dermoid cyst. Further suctioning revealed the presence of thick, chocolate-coloured fluid suggestive of endometriotic contents. The cyst was enucleated and sent for histopathological examination. The right fallopian tube, left ovary, and left fallopian tube were macroscopically normal.

**Figure 2:**
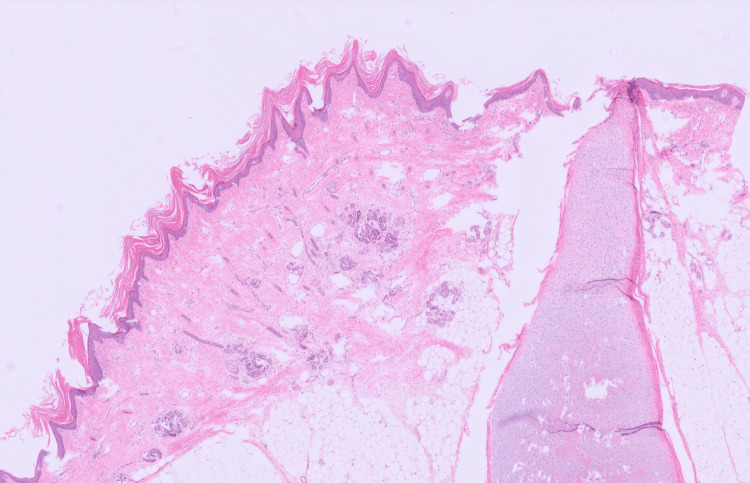
H&E x20 shows skin, including adnexal structure, adipose tissue and cartilaginous tissue

**Figure 3:**
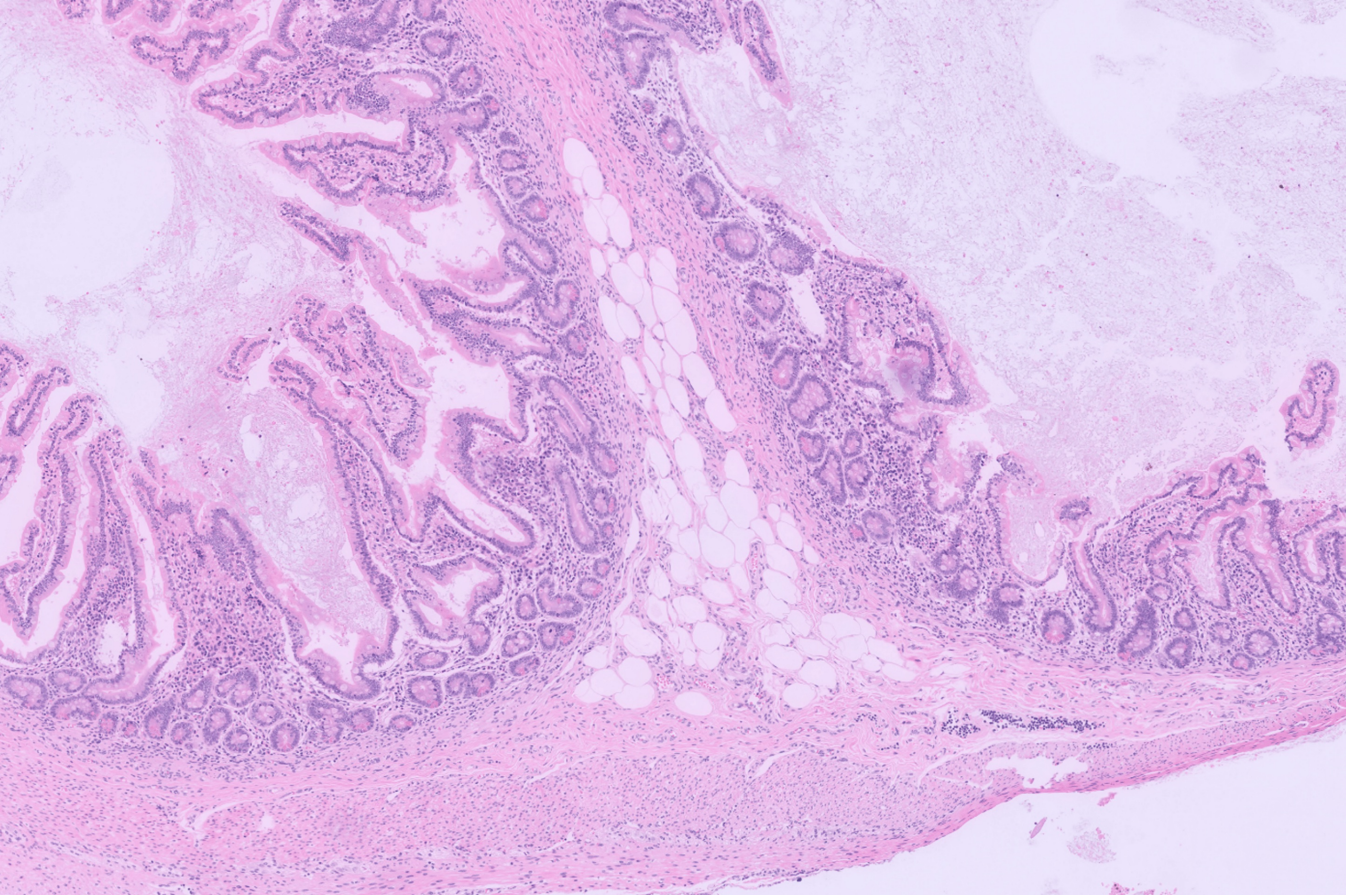
H&E x50 shows intestinal mucosa and adipose tissue

**Figure 4:**
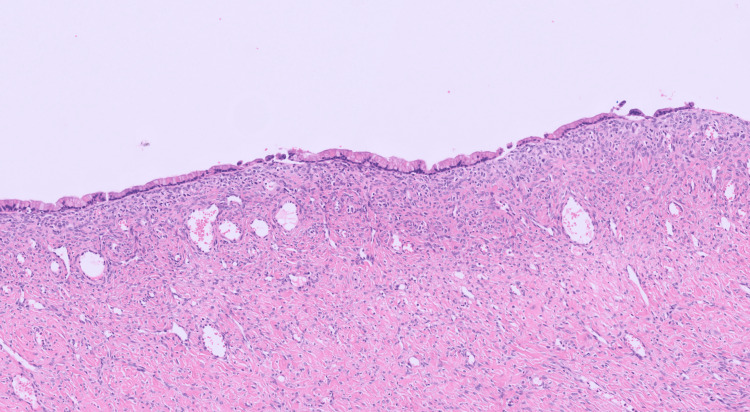
H&E x100 shows mucinous cystadenoma

Histopathological examination confirmed a dermoid cyst measuring 120 mm × 60 mm × 45mm and weighing 120 g. Gross examination of the cyst content demonstrated greasy keratinaceous debris and hair. Sectioning revealed a solid locule measuring up to 50 mm in greatest dimension, containing bone, cartilage, and adipose tissue, and lined by skin-like epithelium with hair (Figures 2, 3). Focal areas of haemorrhage were also noted. Microscopic examination of the haemorrhagic cystic area identified a distinct adjacent cyst lined by attenuated mucinous epithelium overlying ovarian stroma, without architectural complexity, consistent with a benign mucinous cystadenoma (Figure 4). There was no evidence of cytological atypia or malignancy. No malignant cells were identified in the ovarian cyst aspirate. Endometrial curettings demonstrated early secretory phase endometrium with no evidence of hyperplasia, infection, or neoplasia.

Based on the combined radiological, intraoperative, and histopathological findings, a diagnosis of an ovarian collision tumour comprising a mature cystic teratoma and a benign mucinous cystadenoma was made. The patient had an uncomplicated postoperative recovery and remained well on follow-up.

## Discussion

Collision tumours are rare neoplasms characterised by the coexistence of elements of distinct histologic origins within a single organ, with their margins attached to each other but with no significant tissue admixture. Collision tumours of the ovary that have been reported in the literature are mixtures of different combinations of epithelial tumours, germ cell tumours and sex cord stromal tumours. They are predominantly benign, with malignant components reported only in rare, isolated cases. Among the various combinations reported, the most common association involves a mature cystic teratoma with a benign epithelial ovarian tumour, particularly serous or mucinous cystadenoma, as demonstrated in this case report [4-7].

Ovarian germ cell tumours are derived from primordial germ cells of the ovary, with benign mature cystic teratoma (dermoid cyst) being the most common type. These tumours are composed of well-differentiated tissues derived from the ectoderm, mesoderm, and endoderm. Mature cystic teratomas typically occur in women in their second to third decades of life, are usually unilateral, and are bilateral in only 10-17% of cases [8,9]. Epithelial ovarian tumours constitute approximately 60% of all ovarian tumours and 40% of benign ovarian neoplasms. Among them, ovarian cystadenomas are common benign epithelial tumours and include serous, mucinous, endometrioid, and seromucinous types. Benign mucinous cystadenomas represent about 80% of ovarian mucinous tumours, predominantly affect women aged 30 to 60 years, and are unilateral in the majority (95%) of cases [10].

The pathogenesis underlying the coexistence of a mature cystic teratoma and a mucinous cystadenoma remains uncertain. Mature cystic teratomas arise from totipotent germ cells capable of differentiating into tissues derived from all three embryologic germ layers. In contrast, mucinous cystadenomas are epithelial tumours believed to originate from the ovarian surface epithelium or müllerian-type epithelium. One proposed mechanism suggests that mucinous tumours may arise from mucinous or gastrointestinal-type epithelium present within a teratoma, as such epithelium is commonly identified in dermoid cysts. However, in true collision tumours, the two neoplasms remain histologically distinct without histological admixture, suggesting independent tumourigenesis rather than derivation from a single precursor lesion. Alternatively, their coexistence may be coincidental, given that both tumours are among the most common benign ovarian neoplasms. Another hypothesis is that the presence of the first tumour changes the microenvironment, such as angiogenesis and inflammation, leading to the development of the second adjacent tumour [4,11,12]. Regardless of the underlying mechanism, recognition of this entity is clinically important, as management and prognosis are determined by the most aggressive component.

Ovarian collision tumour occurs most frequently in middle-aged women. A case series by Peng et al. reported the average age of diagnosis was 40 years (23 to 67 years) [13]. The clinical presentation of an ovarian collision tumour is typically non-specific. Patients can complain of abdominal pain and distension along with pressure symptoms due to mass effects from the tumour such as an increase in urinary frequency. Dysfunctional uterine bleeding and irregular menstruation can happen, but less commonly. An elevated CA-125 level is the most common finding from the laboratory examinations, suggesting an epithelial component to the ovarian collision tumour [4].

Radiological examination, such as ultrasound, CT and magnetic resonance imaging (MRI), plays an important role in providing information about the overall tumour and clear differentiation of each component, aiding preoperative diagnosis of an ovarian collision tumour. Ultrasound is often the initial imaging modality for adnexal masses. Ultrasound features of ovarian collision tumours can be grouped into three patterns, though preoperative diagnosis remains challenging. These include: (1) two adjacent but separate masses with clear boundaries and distinct imaging characteristics; (2) a “tumor-within-a-tumor” appearance, where a smaller lesion lies within or inside a larger cystic structure, each with its own features; and (3) a single complex mass with heterogeneous and mixed characteristics that cannot be attributed to a single tumor type [14]. CT and MRI can further characterise these patterns. CT is useful for identifying differences in attenuation, such as fat, calcification, or soft tissue components, while MRI provides superior tissue characterisation and more clearly delineates the coexistence of two distinct tumour components [13,15]. A collision tumour should be considered when an ovarian teratoma has radiological findings that cannot be explained solely by an ovarian teratoma [16].

Ovarian collision tumours often pose a diagnostic challenge due to their rarity and nonspecific clinical and radiological features. Preoperative imaging typically reveals a complex adnexal mass, which may mimic a single neoplasm or raise suspicion for malignancy. As a result, a definitive diagnosis is usually established only after surgical excision and detailed histopathological examination. Preoperative suspicion of an ovarian collision tumour is crucial, as it prompts the pathologists to perform a thorough examination of the tumour as well as biopsies from the appropriate section. Accurate histopathological evaluation is essential to distinguish collision tumours from mixed malignant tumours or metastatic disease. Recognition of the individual tumour components is particularly important, as the management of ovarian malignancy may differ substantially depending on tumour type and stage, and may include additional surgical staging procedures, further surgery, or adjuvant chemotherapy. The presence of an ovarian collision tumour does not inherently increase malignancy risk; treatment decisions and prognosis are typically guided by the histological nature of the individual tumour components.

In this case study, there were some radiological and intraoperative cues regarding the existence of an ovarian collision tumour. However, given the rare reports of ovarian collision tumours and lack of awareness, the presence of a collision tumour was not recognised preoperatively.

## Conclusions

Ovarian collision tumours are rare entities characterised by the coexistence of two histologically distinct neoplasms within the same ovary. The present case highlights the coexistence of a mature cystic teratoma with a benign mucinous cystadenoma, emphasising the diagnostic challenges encountered in preoperative imaging and intraoperative assessment. Definitive diagnosis relies on meticulous histopathological examination to identify and delineate the individual tumour components. Awareness of such rare combinations is essential for clinicians, especially gynaecologists, radiologists, and pathologists, as accurate diagnosis has important implications for appropriate management, prognosis, and follow-up. Reporting rare cases of ovarian collision tumours contributes to a better understanding of their pathogenesis, clinicopathological features, and best treatment strategies.
